# Genetic and Environmental Factors Associated with Laboratory Rearing Affect Survival and Assortative Mating but Not Overall Mating Success in *Anopheles gambiae* Sensu Stricto

**DOI:** 10.1371/journal.pone.0082631

**Published:** 2013-12-31

**Authors:** Doug Paton, Mahamoudou Touré, Adama Sacko, Mamadou B. Coulibaly, Sékou F. Traoré, Frédéric Tripet

**Affiliations:** 1 Centre for Applied Entomology and Parasitology, Institute for Science and Technology in Medicine, Keele University, Staffordshire, United Kingdom; 2 Malaria Research and Training Centre, Faculty of Medicine Pharmacy and Dentistry, University of Sciences, Techniques and Technologies of Bamako, Bamako, Mali; Virginia Tech, United States of America

## Abstract

*Anopheles gambiae* sensu stricto, the main vector of malaria in Africa, is characterized by its vast geographical range and complex population structure. Assortative mating amongst the reproductively isolated cryptic forms that co-occur in many areas poses unique challenges for programs aiming to decrease malaria incidence via the release of sterile or genetically-modified mosquitoes. Importantly, whether laboratory-rearing affects the ability of *An. gambiae* individuals of a given cryptic taxa to successfully mate with individuals of their own form in field conditions is still unknown and yet crucial for mosquito-releases. Here, the independent effects of genetic and environmental factors associated with laboratory rearing on male and female survival, mating success and assortative mating were evaluated in the Mopti form of *An. gambiae* over 2010 and 2011. In semi-field enclosures experiments and despite strong variation between years, the overall survival and mating success of male and female progeny from a laboratory strain was not found to be significantly lower than those of the progeny of field females from the same population. Adult progeny from field-caught females reared at the larval stage in the laboratory and from laboratory females reared outdoors exhibited a significant decrease in survival but not in mating success. Importantly, laboratory individuals reared as larvae indoors were unable to mate assortatively as adults, whilst field progeny reared either outdoors or in the laboratory, as well as laboratory progeny reared outdoors all mated significantly assortatively. These results highlight the importance of genetic and environment interactions for the development of *An. gambiae's* full mating behavioral repertoire and the challenges this creates for mosquito rearing and release-based control strategies.

## Introduction

In the last 20 years, the mass-distribution of insecticide treated nets (ITNs) and large-scale indoor residual spraying (IRS) of insecticides have been effective in reducing the incidence of malaria in a number of endemic countries [1], [2]. Despite these successes, there is a real danger that these achievements could be undone by the fast spread of resistance to insecticides observed in the main malaria vectors, *An. gambiae sensu stricto*, *An. arabiensis* and *An. funestus*. The situation is compounded by the limited availability of chemicals approved for indoor use and the widespread occurrence of cross-target site and metabolic resistance to those compounds in malaria vectors (reviewed in [3], [4]).

The need for development of not only new insecticides, but also novel and alternative approaches to vector control explains the renewed interest in and rapid expansion of research focused on vector control using either sterile male releases [5] or the release of genetically-manipulated mosquitoes unable to transmit malaria [6]. Underpinning these approaches is the requirement to consistently raise, sort and release large numbers of sexually competitive male mosquitoes to target wild vector populations [7], [8]. This presents a number of challenges, particularly when those populations have complex population structures and vast geographical ranges, as is the case for some of most important malaria vectors in Africa [9], [10], [11]. In the case of *An. gambiae* s.s., the most relevant sibling species of the *An. gambiae* complex in terms of abundance and level of anthropophily, the known presence of sympatric cryptic taxa in many regions combined with the current poor knowledge of processes leading to assortative mating over the majority of its geographical range casts doubt on the feasibility of implementing release projects [12]. Thus it has become imperative to further our understanding of the environmental, genetic and behavioral processes that determine, not only the competitiveness of mass-produced and released individuals, but also their mating choosiness.

Notwithstanding the direct fitness effects of sterilisation or transgenesis [13], [14], one of the primary factors affecting the competitiveness and fitness of a release-candidate strain is that of the effect of colonisation [7], [15]. During the process of establishing a new laboratory colony, the mosquito population undergo at least one, and possibly several selective sweeps and genetic bottlenecks as the newly colonised strain adapts to insectary conditions. As an example, Norris and colleagues [16] reported an 8-fold decrease in allelic richness, and a 3.5 fold decrease in heterozygosity in laboratory populations of *An. gambiae s.s.* when compared to field samples. As the colonised strain adapts to its new environment there is also a strong possibility that it will develop aberrant swarming and mating behaviour in response to new environmental conditions [17], [18]. Differences in light:dark cycle and lack of a crepuscular transition typically employed in insectaries are known to significantly affect the time of male swarming behaviour and female mate-seeking behaviour - both important determinants of mating success in anophelines [19], [20]. In addition, in members of the *An. gambiae* species complex, the lack of a natural horizon or swarm markers in laboratory enclosures could contribute to the divergence in mating behaviour between colonised strains and the wild populations they are derived from [18].

Strong experimental evidence that these factors effectively hinder mating between lab and field mosquito populations - particularly outside of the laboratory - is currently scant because of the complexity of setting up field releases or semi-field experiments in large outdoor cages. In *Culex tritaeniorhynchus*, reduced mating performance was observed during a large-scale sterile-male release in India in 1977 [21]. Crucially, attempts at increasing mating performance by extensive pre-release outcrossing with the progeny of field-caught gravid female *C. tritaeniorhynchus* did not restore their mating phenotype [22]. In Malaysia, Lee and colleagues conducted fully contained large enclosures experiments to assess the mating competitiveness of two RIDL strains of *Aedes aegypti* with a Malaysian or Mexican genetic background, expressing a repressible dominant lethal gene, successfully competed with males from a local wild-type laboratory strain of *Ae. aegypti* [23]. However, the same RIDL males released in open-field trials on Grand Caiman Island, exhibited a 44% reduction in mating competitiveness compared to wild males [24]. Recent studies of the mating competitiveness of the OX3604C transgenic sterile strain of *Ae. aegypti* carrying a dominant female/lethal system showed that males were competitive in large-cage experiments set in the laboratory[25]. However, the same males only achieved up to 59.1% of the competitiveness of wild-type males in semi-field enclosures despite outcrossing the transgenic line to a laboratory strain genetically similar to the target population [26]. As a result male releases were unable to suppress their experimental target populations [26].

The 1970–80s releases of sterile *An. albimanus* in El Salvador and translocation-carrying *An. culicifacies* shed some light on released male competitiveness in anophelines [27], [28]. In *An. albimanus*, chemosterilized males that were shown to be as competitive as non-sterilized males in cage experiments were estimated to have 25% of the mating competitiveness of wild males in release situations [29]. In Lahore, Pakistan, translocation-bearing sterile males failed to mate with wild females because they were reared with a photoperiod that did not match that of the wild target population [20], [27]. In a more recent study in the Sudan, Hassan and colleagues, working in large semi-field enclosures, showed that radio-sterilised *An. arabiensis* males produced from the 68^th^ generation of a laboratory strain were able to compete with non-sterile males produced from field-caught larvae and pupae for wild virgin females. The researchers estimated the competitiveness of the sterile strain as 71% that of their non-sterile counterparts [30]. These examples emphasize the complex interplay of genetic and environmental factors on mating performance of laboratory-reared individuals.

There are currently no published field-based studies on the mating competitiveness of laboratory-reared *An. gambiae* s.s. versus that of wild individuals from potential target populations. This comes as a surprise given the extensive research effort undertaken in the last 30 years in order to improve our understanding of *An. gambiae*'s complex population structure and speciation processes. Within the sibling species *An. gambiae s.s*., 5 chromosomal forms known as Mopti, Savanna, Bamako, Forest and Bissau have been characterized through typical inversion polymorphisms on the 2R chromosome [31], [32], [33], [34]. In addition, two molecular forms exhibiting fixed sequence differences in the intergenic spacer of the ribosomal DNA on the X chromosome and referred to as M and S molecular forms have been identified [35], [36], [37]. The combination of these two marker types currently defines 8 cryptic taxa that vary in geographical distribution and habitat use [33]. The level of reproductive isolation between these sub-populations is currently debated and a major taxonomic revision aiming to elevate the M molecular form to sibling species status is currently considered [38]. Here, the Mali Mopti chromosomal populations characterized by M-type rDNA and the *bc* and *u* inversions arrangements are referred to as Mopti form throughout the text whilst the Savanna and Bamako form refer to those characterized by S-type rDNA and high frequencies of *b*, *cu*, *bcu* and *jbcu*, *jcu* arrangements respectively [32].

Importantly, these sub-taxa sometimes also differ in how difficult they are to colonize in the laboratory. As an example, populations of the Mopti form from Mali adapt readily to mating, feeding and laying eggs under insectary conditions. However, the Bamako and Savanna forms that co-occur with the Mopti form in large parts of the country are much harder to colonize (FT Pers. Obs.). A direct corollary from those observations is that hard-to-colonize chromosomal forms undergo stronger selection and bottle-necks in the colonization process and will ultimately require complex out-crossing schemes to regain a wild-type like mating phenotype prior to being released [8]. Another complication stems from the fact that colonized cryptic taxa readily cross-mate under laboratory conditions whilst in the wild they are separated by strong pre-mating reproductive barriers [12], [39]. These observations emphasize the task at hand for vector control strategies aiming to release individuals mating both competitively and assortatively.

Ensuring that laboratory-reared individuals mate assortatively is further compounded by our poor understanding of the behavioral processes leading to strong assortative mating amongst wild sympatric populations [39]. Assortative mating is thought to result from several potential processes, including spatial swarm segregation [40], the recognition of specific flight tones [41], [42], and potential recognition through vision and olfaction [34]. However, there is no consensus over the relative importance of these processes and, a fortiori, on how to preserve their integrity during mosquito colonization, mass-rearing and releases.

The selective pressure associated with the colonization process, the underlying complexity of the processes involved in mate recognition in the wild, and the contrasting levels of assortative mating observed between colonized and wild strain, suggest that a full mating-behavioural repertoire may strongly depend on the genetic quality of released individuals. However, there are also indications that mating behaviour may be affected by insectary-rearing independently of direct effects of genetic quality. For example, differences in dark:light cycle between the laboratory and the field were shown to dramatically affect mating competitiveness in releases of *An. culicifacies* [20], but it is unclear if that pattern was due to selection for a different photoperiod or a phenotypic plastic response to it. Interestingly, Dao et al. [43] showed in a recent study that laboratory-reared progeny of field-collected sympatric M and S form females cross-mated at much higher then expected frequency when release inside huts. This finding suggests that laboratory conditions experienced at the larval stage might negatively impact cross-mating avoidance at the adult stage - at least in the hut environment. Whether the same phenomenon occurs when mating takes place outdoors has not yet been explored despite important ramifications for mosquito release projects.

In order to distinguish genetic effects linked to laboratory-rearing from phenotypic responses affecting the mating behavior of the Mopti form, large-outdoor cages experiments were conducted in 2010 and 2011 in the village of N'Gabacoro droit, Mali, West Africa. The larval progeny of females either field-caught or from a recently colonized strain of the local Mopti form were reared either in the insectary or outdoors using otherwise identical rearing techniques. This experimental design enabled us to assess the relative importance of genetic and environmental effects associated with laboratory-rearing affecting male and female survival and mating success as well as their ability to choose between mates of their own and a different sympatric sub-taxa. The impacts of genetic and environment factors associated with laboratory breeding on the mating behavioural repertoire of this important vector species and their implications for future mosquito-release projects are discussed.

## Results

### 1st experiment - Genetic/Environmental effects on survival and mating success within form

In the first experiment conducted in Aug–Sept 2010 and 2011, Mopti form males and females from each of the 4 treatment groups (genetic/environmental background respectively: Field/Field, Field/Lab, Lab/Field and Lab/Lab) were mixed with an equal number of Mopti form Field/Field mosquitoes of the opposite gender in field mating enclosures.

#### Body Size

**Figure 1:**
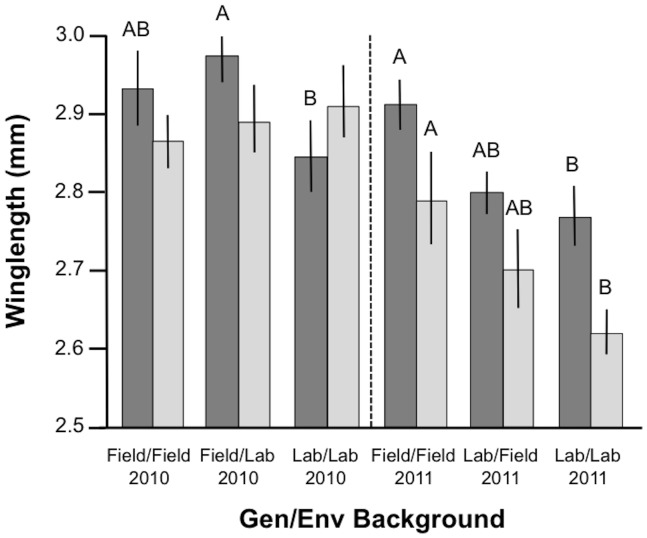
Mean wing length (mm) in females (dark bars) and males (light bars) from the 4 genetic/environmental groups in the 2010–2011 within-form mating experiment. For each gender, levels labelled with different letters differed significantly in pairwise statistical comparisons (Tukey test). Error bars are 95% confidence intervals.

Two general linear models indicated that despite standard larval rearing protocols, females and males from the 4 Gen/Env experimental treatments were significantly larger in 2010 than in 2011 (Table 1). Mean female wing length was equal to 2.91 mm (2.89–2.92CI) in 2010 and 2.84 mm (2.82–2.85) in 2011 and, respectively, 2.92 mm (2.90–2.94) and 2.73 mm (2.71–2.75) in males. A significant amount of the variance in wing length in both females and males was also explained by replication within the experiment. In both females and males there were significant differences in size between individuals from the 4 experimental Gen/Env treatments and a significant interaction between replicate and experimental treatment (Table 1, Fig. 1). Breaking down this analysis per year revealed a significant direct effect of Gen/Env treatment on female body size and an interaction with replicate in 2010 (General linear model: Gen/Env: *F*_2,229_ = 3.96, *P* = 0.020, Gen/Env * replicate: *F*_4,229_ = 2.60, *P* = 0.038) but not in males (General liner model: Gen/Env: *F*_2,152_ = 0.47, *P* = 0.624, Gen/Env*replicate: *F*_4,152_ = 1.12, *P* = 0.332). Pairwise comparisons of least-square means indicate that Field/Lab females were significantly larger than Lab/Lab ones (Tukey: *P* = 0.015) whilst the other two comparisons were not significant (Tukey: *P*>0.05 in both cases)(Table 2).

**Table 1:** General linear models of the effects of Experimental Year, Replicate, and Genetic/Environmental treatment on female and male body size in the within-form mating experiment (1^st^ experiment).

		Females*		Males*	
Source†	DF	*F*-ratio	*P*	*F*-ratio	*P*
Year	1	47.72	<0.001	112.56	<0.001
Replicate[Year]	4	18.06	<0.001	7.21	<0.001
Gen/Env[Year]	4	9.78	<0.001	5.33	<0.001
Replicate*Gen/Env[Year]	8	4.54	<0.001	2.18	0.029

Square brackets indicate effect nesting.

Sample sizes were 469 females and 304 males.

**Table 2:** Mean female and male body size, survival and insemination rate in relation to Genetic/Environmental treatment in the within-form mating experiment (1^st^ experiment).

	Body size (mm)		Survival rate (%)		Insemination rate (%)	
Gen/Env Treatment	Females	Males	Females	Males	Females§	Males†
Field/Field 2010	2.94 (2.90–2.98)	2.87 (2.83–2.90)	66.0 (58.1–73.1)	52.7 (44.7–60.5)	30.9 (22.6–40.7)	38.8 (29.7–48.7)
	*95*	*75*	*99/150*	*79/150*	*30/97*	*38/98*
Field/Lab 2010	2.98 (2.94–3.01)	2.90 (2.83–2.90)	48.7 (40.8–56.6)	41.3 (33.8–49.3)	16.9 (9.9–27.2)	25.0 (17.3–34.7)
	*73*	*44*	*73/150*	*62/150*	*12/71*	*23/92*
Lab/Lab 2010	2.84 (2.80–2.89)	2.91 (2.87–2.96)	48.0 (40.1–55.9)	29.3 (22.6–37.0)	22.9 (14.6–34.0)	21.4 (14.0–31.3)
	*70*	*42*	*72/150*	*44/150*	*16/70*	*18/84*
Field/Field 2011	2.92 (2.88–2.94)	2.79 (2.74–2.85)	54.0 (46.0–61.8)	34.0 (26.9–41.9)	31.2 (21.9–42.2)	33.0 (24.4–42.8)
	*81*	*51*	*81/150*	*51/150*	*24/77*	*32/97*
Lab/Field 2011	2.80 (2.77–2.82)	2.70 (2.65–2.75)	44.7 (36.9–52.7)	24.7 (18.5–32.1)	16.4 (9.4–27.1)	27.1 (16.6–41.0)
	*67*	*35*	*67/150*	*37/150*	*11/67*	*13/48*
Lab/Lab 2011	2.77 (2.73–2.81)	2.62 (2.58–2.66)	55.3 (47.3–63.1)	36.7 (29.4–44.7)	16.9 (10.5–26.0)	38.4 (26.5–52.0)
	*83*	*57*	*83/150*	*55/150*	*15/89*	*20/52*

The insemination rate of females of each treatment exposed to Field/Field Mopti males.

Here the insemination rate of Field/Field Mopti females exposed to males of each treatment group.

Values in brackets are 95% confidence intervals and sample sizes are indicated in italics.

In 2011, there were significant direct effects of Gen/Env treatment on female body size and interaction with replicate in both genders (General liner model: Females Gen/Env: *F*_2,222_ = 19.2, *P*<0.001, Gen/Env*replicate: *F*_4,222_ = 7.72, *P*<0.001; Males Gen/Env: *F*_2,134_ = 9.21, *P*<0.001, Gen/Env*replicate: *F*_4,134_ = 2.99, *P* = 0.021). Pairwise least-square means comparisons showed that both females and males from the Field/Field group were significantly larger than the Lab/Field and Lab/Lab ones (Tukey: females *P*<0.001 and males *P*<0.01 in both cases) but that the later two groups did not differ between one another in either sex (Tukey: females *P* = 0.365, males *P* = 0.816)(Fig. 1).

The Field/Field Mopti females and males that were paired with treatment females and males in the field mating enclosures had a mean wing length of 2.89 mm (2.87–2.91) and 2.94 mm (2.92–2.97) in 2010 and respectively, 2.84 mm (2.82–2.87) and 2.76 mm (2.74–2.78) in 2011.

#### Survival

Of the 1800 female and 1800 male mosquitoes released as part of this experiment, we recaptured 960 females (53.3%) and 705 males (39.2%) after two nights in the enclosures equivalent to daily survival rates of 73.0 and 62.6%.

Female and male survival was analyzed using logistic regression models (Table 3). Average male survival rate was equal to 41.1% (36.7–45.7) in replicates conducted in 2010 and significantly higher than in the 2011 replicates where survival was 38.2% (27.6–36.2). There was no significant year effect in females, with an average survival of 54.2% (49.6–58.8) in 2010 and 51.3% (46.7–55.9) in 2011.

**Table 3:** Nominal logistic regressions of the effects of Year, Genetic/Environmental treatment, and Enclosure on female and male survival in within-form mating experiment (1^st^ experiment).

		Females*		Males*	
Source†	DF	LR Chi-Square	*P*	LR Chi-Square	*P*
Year	1	0.64	0.422	8.04	0.005
Gen/Env[Year]	4	18.8	0.002	19.96	<0.001
Enclosure	2	51.82	<0.001	25.08	<0.001
Enclosure*Year	2	6.81	0.033	23.28	<0.001
Enclosure*Gen/Env[Year]	8	56.03	<0.001	99.93	<0.001

Square brackets indicate effect nesting.

Sample sizes were 900 females and 900 males.

**Figure 2:**
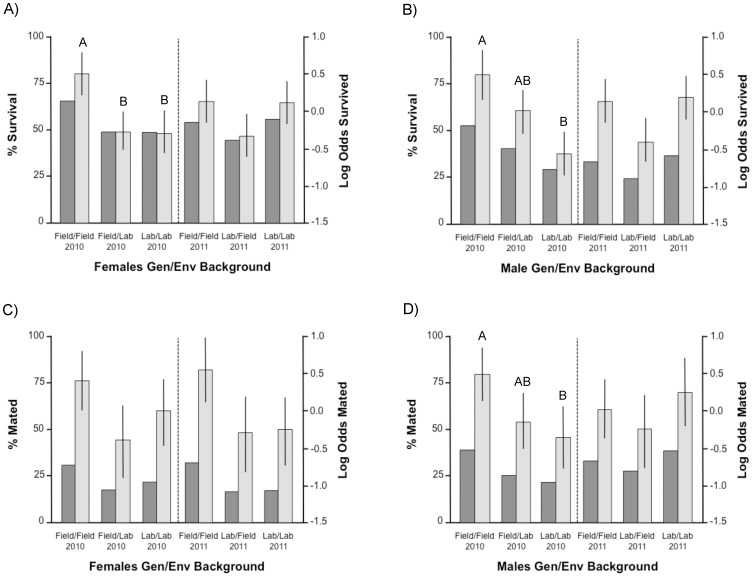
Percentages (dark columns and left axis) and Log Odds estimates (light columns and right axis) of survival and mating rates in males and females from 4 combinations of genetic/environmental backgrounds after 2 nights under semi-field conditions in the 2010–2011 within-form mating experiment. A–B: Survival of treatment females and males; C–D: Mating rate of treatment females and males. For each gender, levels labelled with different letters differed significantly in pairwise statistical comparisons (Tukey test). Error bars represent 95% confidence intervals.

In both sexes, there were significant differences in survival over two mating nights in relation to their Gen/Env experimental treatment and the enclosure used, and there were significant interactions between enclosures, year and experimental treatment (Table 3). Breaking down these analyses by year whilst correcting for the effect of enclosure revealed significant differences in female and male survival in 2010 (Logistic regressions: Gen/Env: *χ* ^2^ = 13.9, *P* = 0.001 and *χ* ^2^ = 14.2: *P*<0.001 respectively). Pairwise group comparisons showed that female survival in the Field/Field group was significantly higher than in Field/Lab females and Lab/Lab ones (Marascuilo pairwise comparisons: *χ^2^* = 9.50, *P* = 0.009 and *χ^2^* = 10.3, *P* = 0.006) (Table 2, Fig. 2A). In males, survival in the Field/Field group only tended to be higher than in the Field/lab one (Marascuilo comparison: *χ* ^2^ = 4.80, *P* = 0.091) but was significantly higher than that observed in Lab/Lab males (*χ* ^2^ = 17.9, *P*<0.001)(Fig. 2B). In 2011, there were no significant differences between any of the female or male Gen/Env treatment groups although results were very close to the statistical threshold (Logistic regression: Gen/Env: *χ* ^2^ = 4.19, *P* = 0.123 and *χ* ^2^ = 5.72, *P* = 0.057)(Table 2, Fig. 2A–B).

#### Mating Success

Across the whole of the 2010–2011 experiment, 252 of the 959 females recaptured alive after 2 nights (26.28%) had sperm in their spermatheca. Insemination rates were analyzed using logistic regression models. There was no significant year effect on insemination rates in replicates that focused on the Gen/Env background of females, nor in replicates focusing on males (Table 4). Breaking down the analysis by year revealed that in 2010, there was no significant effect of the Gen/Env treatment in females (Logistic regression: Gen/Env: *χ* ^2^ = 4.56, *P* = 0.102). In 2011, the Gen/Env of females had a significant overall effect on insemination rates (Logistic regression: Gen/Env: *χ* ^2^ = 6.19, *P* = 0.045). However none of the post-hoc pairwise comparisons were significant (Marascuilo comparisons: *P*>0.05 in all cases) (Table 2, Fig. 2C). Male Gen/Env significantly affected their insemination rates (Logistic regression: Gen/Env: *χ* ^2^ = 7.52, *P* = 0.023)(Fig. 2D). Field/Field males inseminated more females than Lab/Lab ones in 2010 (Marascuilo comparison: *χ* ^2^ = 1.17, *P* = 0.033). There was no significant between treatment group differences in 2011 (Logistic regression: Gen/Env: *χ* ^2^ = 1.47, *P* = 0.479)(Table 2, Fig. 2C–D).

**Table 4:** Mean female and male body size, survival and insemination rate in relation to Genetic/Environmental treatment in the assortative mating experiment (2^nd^ experiment).

	Body size (mm)		Survival rate %		Overall insemination rate %	
Gen/Env Treatment	Females	Males	Females	Males	Females§	Males†
Field/Field 2011	2.71 (2.67–2.75)	2.94 (2.87–3.01)	65.3 (57.4–72.3)	35.3 (28.1–43.3)	16.0 (10.1–24.4)	36.3 (26.6–47.2)
	*100*	*47*	*98/150*	*53/150*	*16/100*	*29/80*
Field/Lab 2011	2.86 (2.80–2.92)	2.74 (2.64–2.84)	32.0 (25.1–39.8)	8.00 (4.64–13.6)	22.9 (13.3–36.5)	22.5 (12.3–37.5)
	*48*	*12*	*48/150*	*12/150*	*11/48*	*9/40*
Lab/Field 2011	2.93 (2.87–2.98)	2.73 (2.67–2.79)	28.7 (22.0–36.4)	17.3 (12.1–24.2)	20.5 (11.15–34.5)	25.0 (13.3–42.1)
	*44*	*26*	*43/150*	*26/150*	*9/44*	*8/32*
Lab/Lab 2011	2.65 (2.62–2.68)	2.59 (2.54–2.65)	61.3 (53.3–68.8)	32.0 (25.1–39.8)	18.6 (11.8–28.1)	35.9 (22.7–51.6)
	*92*	*46*	*92/150*	*48/150*	*16/86*	*14/39*

The insemination rate of females of each treatment exposed to Field/Field Mopti males.

Here the insemination rate of Field/Field Mopti females exposed to males of each treatment group.

Values in brackets are 95% confidence intervals and sample sizes are indicated in italics.

### 2nd Experiment - Genetic/Environmental effects on survival and assortative mating

In the second experiment conducted in Sept–Oct 2011, males and females Mopti form from each of the 4 treatment groups (genetic/environmental background respectively: Field/Field, Field/Lab, Lab/Field and Lab/Lab) were mixed with an equal number of a 1∶1 mix of Mopti form and Savanna form Field/Field mosquitoes of the opposite gender in the field mating enclosures.

#### Body Size

**Figure 3:**
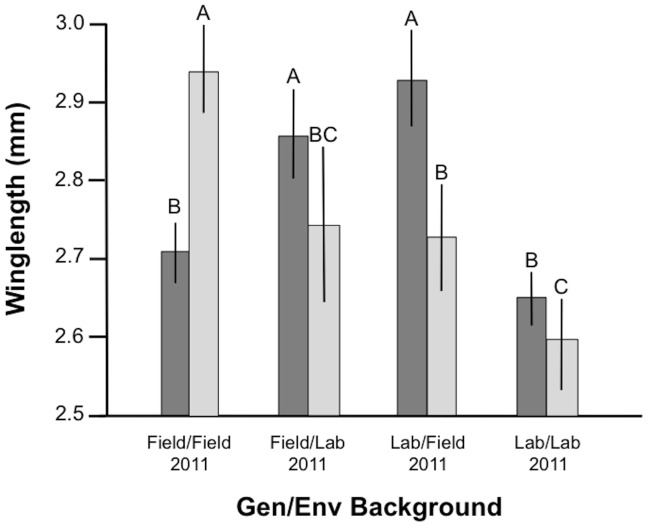
Mean wing length (mm) in females (dark bars) and males (light bars) from the 4 Genetic/Environmental treatments in the 2011 assortative mating experiment. For each gender, levels labelled with different letters differed significantly in pairwise statistical comparisons (Tukey test). Error bars are 95% confidence intervals.

The average body size in the 2nd experiment across all 4 experimental groups was 2.75 mm (2.73–2.78) for females and 2.76 mm (2.72–2.80) in males. There was no significant effect of replicates on body size in the second experiment. The Gen/Env experimental treatments significantly affected body size in females (ANOVA: *F*_3,280_ = 28.1, *P*<0.001) as well as in males (ANOVA: *F*_3,127_ = 23.1, *P*<0.001). In females, the Field/Lab and Lab/Field treatment groups were significantly larger than both the Field/Field and Lab/Lab cohorts (Tukey: *P*<0.001 in both cases)(Table 4, Fig. 3). In males, individuals from the Field/Field group were significantly larger than those in other groups (*P*<0.013 in all cases). The Lab/Field group was significantly larger than Lab/Lab individuals (Tukey: *P* = 0.034) but did not differ from Field/Lab ones (Tukey: *P* = 0.999). Field/Lab males did not differ from Lab/Lab ones, largely because of the large variance in this group (Tukey: *P* = 0.119)(Table 4, Fig. 3).

The Field/Field Mopti and Savanna females that were paired with treatment males and females in mating enclosures had a mean wing length of 2.92 mm (2.89–2.96) and 2.93 mm (2.90–2.96) and did not differ significantly (*T*-test: df = 188, *t*-ratio = 0.32, *P* = 0.747). Similarly, there was no difference between Field/Field Mopti and Savanna males of size 2.86 mm (2.81–2.90) and 2.90 mm (2.85–2.97)(*T*-test: df = 99, *t*-ratio = 1.45, *P* = 0.150).

#### Survival

**Figure 4:**
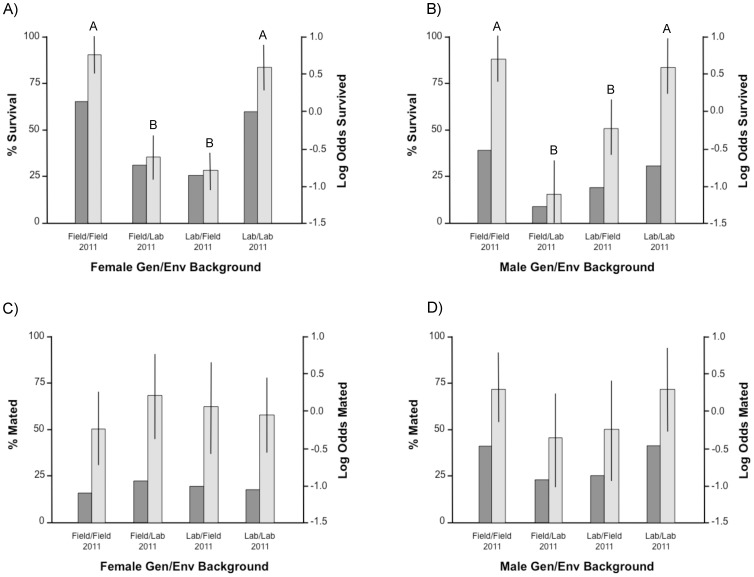
Percentages (dark columns and left axis) and Log Odds estimates (light columns and right axis) of survival and mating rates in males and females from the 4 Genetic/Environmental treatments after 2 nights in the 2011 assortative mating experiment. A–B: Survival rate of treatment females and males; C–D: Mating rate of treatment females and males. For each gender, levels labelled with different letters differed significantly in pairwise statistical comparisons (Tukey test). Error bars represent 95% confidence intervals.

Of the 1200 females and 1200 males released in the semi-field enclosures, we recaptured 479 females (39.9%) and 223 males (18.6%) after two nights equivalent to daily survival rates of 63.2 and 43.1%. There was no significant effect of enclosures on survival in the second experiment. In females, Gen/Env treatment significantly affected survival as well as replicate (Logistic regression: Gen/Env: *χ* ^2^ = 68.5, *P*<0.001; replicate: *χ* ^2^ = 6.08, *P* = 0.048). Pairwise comparisons show that the survival of Field/Field M females did not significantly differ from that of Lab/Lab ones (Marascuilo comparison: *χ* ^2^ = 0.52, *P* = 0.915) but that the survival of both groups was significantly higher than that of Field/Lab and Lab/Field females (Marascuilo comparisons: *P*<0.001 in all cases). There was no statistical difference between Field/Lab and Lab/Field females (*χ* ^2^ = 0.40, *P* = 0.941) (Table 4, Fig. 4A).

In males, Gen/Env experimental treatment had a significant effect on survival but there was no effect of replicate (Logistic regression: Gen/Env: *χ* ^2^ = 44.9, *P*<0.001; replicate: *χ* ^2^ = 3.40, *P* = 0.1824). There was no significant difference in survival between Field/Field and Lab/Lab males (Marascuilo comparison: *χ* ^2^ = 0.37, *P* = 0.946) and males from both groups survived significantly better than Field/Lab and Lab/Field ones (Marascuilo procedure: *P*<0.05 for all 4 comparisons). There was no statistical difference between Field/Lab and Lab/Field males (*χ* ^2^ = 6.02, *P* = 0.110) (Table 4, Fig. 4B).

In Field/Field males mated to females from the 4 treatment groups, 52/248 M-form males or 17.3% (13.5–22.0) survived compared to 32/268 S-form males or 10.6% (7.66–14.7)(Chi-square: *χ* ^2^ = 5.54, *P* = 0.017). Amongst Field/Field females mated to treatment males, 131/315 M-form females survival was equivalent to 41.6% (36.3–47.1) and this was significantly higher than the 67/285 or 23.5% (19.0–28.8) surviving S-form females (Chi-square: *χ* ^2^ = 22.2, *P*<0.001).

#### Mating Success

In total, we recovered 104 sperm bundles from the spermathecae of 479 surviving females (21.7%). The determinants of overall mating rate were investigated using logistic regressions. There was no effect of replicate or enclosure on female and male mating rate in the second experiment. Neither was there an effect of Gen/Env treatment on mating rate in replicates focusing on the effect of treatment on females (Logistic regression: df = 3, *χ* ^2^ = 1.12, *P* = 0.772) or on those focusing on males (Logistic regression: df = 3, *χ* ^2^ = 0.0, *P* = 0.988)(Table 4, Fig. 4C–D).

#### Assortative Mating Behaviour

Finally, we assessed the degree of assortative mating behaviour exhibited by each of the Gen/Env treatment groups in each sex by determining the molecular form of sperm recovered from mated females (replicates focusing on females) or directly from mated females (replicates focusing on males). Across both genders, there were 74 assortative mating events and 30 disassortative ones (Table 5). We constructed a linear regression model to assess the factors determining variation in the proportion of individuals mating assortatively. There were no significant effects of replicate and enclosure on the proportion of intra-form mating and inter-form mating. Assortative mating was stricter among females than males and varied according to Gen/Env experimental treatment (Logistic regression: Sex: df = 1, *χ* ^2^ = 4.81, *P* = 0.028; Gen/Env: df = 3, *χ* ^2^ = 11.13, *P* = 0.011; interaction NS). In treatment females, 35/44 or 79.5% (65.5–88.8) of sperm bundles were intraspecific, whilst 39/60 or 65% (52.4–75.8) females mated by treatment males were intraspecific (Table 5).

**Table 5:** Intra and inter-form insemination rates with Mopti form and Savanna mates in relation to the Genetic/Environmental treatment of Mopti females and males in the assortative mating experiment (2^nd^ experiment).

	Insemination rate by form %					
Sub-taxa	Mopti (intra-form)			Savanna (inter-form)		
Gen/Env Treatment	Females§	Males†	All	Females§	Males†	All
Field/Field 2011	87.5 (64.0–96.5)	75.9 (57.9–87.8)	80.0 (66.2–89.1)	12.5 (3.50–36.0)	24.1 (12.2–42.1)	20.0 (10.9–33.8)
	*14/16*	*22/29*	*36/45*	*2/16*	*7/29*	*9/45*
Field/Lab 2011	100 (56.6–100)	66.7 (35.4–87.9)	78.6 (52.4–92.4)	0	33.3 (12.1–64.6)	21.4 (7.57–47.6)
	*5/5*	*6/9*	*11/14*	*0/5*	*3/9*	*3/14*
Lab/Field 2011	100 (64.6–100)	62.5 (30.6–86.3)	80.0 (54.8–93.0)	0	37.5 (13.7–69.4)	20 (7.04–45.2)
	*7/7*	*5/8*	*12/15*	*0/7*	*3/8*	*3/15*
Lab/Lab 2011	56.3 (33.2–76.9)	42.9 (21.4–67.4)	50.0 (33.2–66.8)	43.8 (23.1–66.8)	57.1 (32.6–78.6)	50.0 (33.2–66.8)
	*9/16*	*6/14*	*15/30*	*7/16*	*8/14*	*15/30*

The insemination rate of females of each treatment exposed to Field/Field Mopti males.

Here the insemination rate of Field/Field Mopti and Savanna females exposed to males of each treatment group.

Values in brackets are 95% confidence intervals and sample sizes are indicated in italics.

**Figure 5:**
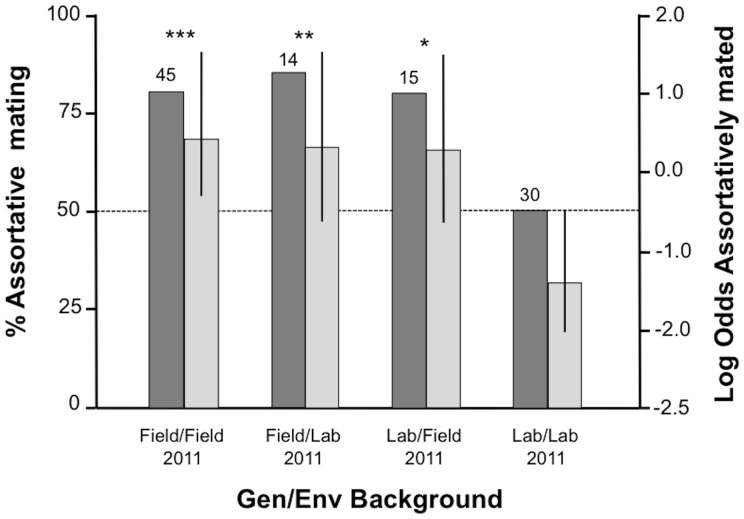
Percentage (dark columns and left axis) and Log Odds estimates (light columns and right axis) of assortative mating after 2 nights under semi-field conditions (both genders combined). Males and females from the 4 experimental groups were exposed to a mixture of M and S mates (combined data shown here). Deviations from a 50∶50 ratio were tested by Chi-square goodness of fit tests (significance values are *P*<0.05 *, *P*<0.01 **, *P*<0.001 ***).

Across both gender significant assortative mating (Mopti to Savanna mating ratio higher than 1∶1) was detected in the Field/Field (Chi-square: df = 1, *χ* ^2^ = 17.34, *P*<0.001), Field/Lab (*χ* ^2^ = 4.86, *P* = 0.028), and Lab/Field (*χ* ^2^ = 5.78, *P* = 0.016). However, there was no evidence of significant assortative mating in the Lab/Lab treatment group (Chi-square: df = 1, *χ* ^2^ = 0, *P* = 1.000)(Fig. 5). We did not observe any instances of multiple mating. Unfortunately, small sample sizes precluded the same analysis to be performed by sex, except for the Field/Field groups. Field/Field females mated significantly assortatively (Chi-square: df = 1, *χ* ^2^ = 9.00, *P* = 0.003) but Lab/Lab ones did not (Chi-square: df = 1, *χ* ^2^ = 0.25, *P* = 0.617). In addition the few mated Field/Lab and Lab/Lab females recaptured alive were all assortatively mated, and this in itself is very improbable (*P*<0.05 in both cases). In males, the Field/Field group significantly deviated from random mating (Chi-square: df = 1, *χ* ^2^ = 7.76, *P* = 0.053) whilst Lab/Lab males did not (Chi-square: df = 1, *χ* ^2^ = 0.29, *P* = 0.593).

The possibility that the lower overall survival of the S-form Field/Field individuals mated to treatment females and males contributed to the observed patterns of assortative mating was further investigated. Although there were significant differences in survival of S-form individuals between treatment groups despite all individuals belonging to the same Field/Field rearing cohort in females (Chi-square: df = 1, *χ* ^2^ = 22.6, *P*<0.001) and males (Chi-square: df = 1, *χ* ^2^ = 27.7, *P*<0.001), patterns of Savanna survival (Table 6) did not suggest they might explain the frequency of disassortative mating. S-form individuals mated with Field/Field treatment males had the highest survival (Table 6), yet most mating events were assortative in this group (Table 6, Fig. 5). Similarly, the Savanna mates of Lab/Lab group had the second lowest survival rate of all groups despite contributing to the highest proportion of disassortative mating (Table 5–6, Fig. 5). Furthermore, the proportion of surviving Mopti to Savanna males and females mated to treatment females and males did not correlate with the proportion of Mopti to Savanna form inseminations in either sex (Spearman correlation: females: n = 12, *ρ* = −0.227, *P* = 0.477; males: n = 12, *ρ* = −0.382, *P* = 0.220).

**Table 6:** Survival rate of Field/Field Mopti form and Savanna form females and males mated to individuals of the 4 Gen/Env treatment groups in the assortative mating experiment (2^nd^ experiment).

	Survival rate by form %			
Sub-taxa	Mopti (intra-form)		Savanna (inter-form)	
Gen/Env Treatment	Females	Males	Females	Males
Field/Field 2011	64.0 (52.7–73.9)	16.0 (9.40–25.9	42.7 (32.1–53.9)	24.0 (15.8–34.8)
	*48/75*	*12/75*	*32/75*	*18/75*
Field/Lab 2011	36.4 (27.1–46.8)	12.0 (6.44–21.3)	12.9 (6.69–23.4)	0
	*32/88*	*9/75*	*8/62*	*0/75*
Lab/Field 2011	20.8 (13.2–31.1)	30.7 (21.4–41.8)	21.9 (14.0–32.7)	14.7 (8.39–24.4)
	*16/77*	*23/75*	*16/73*	*11/75*
Lab/Lab 2011	46.7 (35.8–57.8)	10.7 (5.50–19.7)	14.7 (8.40–24.4)	4.00 (1.14–11.1)
	*35/75*	*8/75*	*11/75*	*3/75*

Values in brackets are 95% confidence intervals and sample sizes are indicated in italics.

### Overall Genetic/Environmental effects on survival and mating success

Since each experiment used a balanced design in terms of the enclosures used and given the high number of replicates conducted, we also performed a general analysis on the pooled data across both experiments in order to examine survival and mating success in relation to the 4 treatments groups with the highest possible statistical power (3000 individuals and 9 replicates). In females, was there was a strong direct effect of treatment on female survival across all replicates (Chi-square: n = 1500, df = 3, *χ* ^2^ = 61.7, *P*<0.001). Pairwise comparisons of Field/Field and Lab/Lab females revealed no difference in survival between the two groups, 61.8 and 54.9% (78.6 and 74.1 daily survival) respectively (Marascuilo comparison: *χ* ^2^ = 4.41, *P* = 0.220). Field/Field and Lab/Lab females survived significantly better than Field/Lab and Lab/Field, 40.3% and 36.7% (63.5 and 60.6 daily survival) (*P*<0.001 in both cases), whilst the later two groups did not differ significantly (*χ* ^2^ = 0.85, *P* = 0.837).

In males there was also a significant effect of treatment on survival (Chi-square: n = 1500, df = 3, *χ* ^2^ = 39.8, *P*<0.001). Pairwise comparisons of Field/Field and Lab/Lab males revealed no difference in survival between the two groups, 40.7% and 32.7% (63.8 and 57.2 daily survival) (Marascuilo comparison: *χ* ^2^ = 6.24, *P* = 0.100). Field/Field males survived significantly better than Field/Lab and Lab/Field ones, 24.7% and 21.0% (63.5 and 60.6 daily survival) (*P*<0.001 in both cases) whilst the later two groups did not differ significantly (*χ* ^2^ = 0.85, *P* = 0.837). Lab/Lab males survived better than Lab/Field ones (*χ* ^2^ = 13.1, *P* = 0.005) but not than Field/Lab ones (*χ* ^2^ = 5.78, *P* = 0.123).

No significant effect of experimental treatment on female mating success (Chi-square: n = 749, df = 3, *χ* ^2^ = 4.58, *P* = 0.205) or in male mating success (Chi-square: n = 662, df = 3, *χ* ^2^ = 7.02, *P* = 0.071) was found across all replicates. Overall, 25.6 Field/Field, 19.3 Field/Lab, 18.2 Lab/Field and 19.2% Lab/Lab females were inseminated (13.7, 10.2, 9.5 and 10.1% insemination rates per night). For the same treatment groups, males inseminated females at rates of 36.0, 24.2, 26.3 and 29.7% (20.0, 13.0, 14.1 and 16.8% nightly insemination rates). The mean mating rate per night for females was 11.3% and males inseminated an average of 16.8% of females.

Across all replicates and treatment groups - i.e. taking each experimental combinations of each replicate as a statistical unit - mean female body size was found to negatively correlated with mean female survival (*n* = 60, *r* = −0.357, *P* = 0.005). In males, there was not significant relationship between their mean body size of males and their mean survival (*n* = 60, *r* = 0.073, *P* = 0.578). The average female mating rate did not significantly correlate with their mean survival (*r* = −0.024, *P* = 0.855) or mean body size (*r* = 1.633, *P* = 0.213). However female insemination rates strongly positively correlated with average male survival (*r* = 0.339, *P* = 0.008) but not mean male body size (*r* = −0.217, *P* = 0.097).

## Discussion

This study reports the first comparisons of survival and mating success of female and male individuals of laboratory strain of the Mopti form of *An. gambiae* s.s. versus wild individuals from their population of origin in semi-field conditions. Replicates conducted in 2010, indicated that Field/Field females and males outperformed Lab/Lab individuals in terms of survival and mating performance. In addition, an experimental group consisting of field progeny reared in the laboratory at the larval stage (Field/Lab group) displayed intermediate survival and mating success. In further replicates conducted in 2011, Field/Field individuals did not outperform Lab/Lab ones. Laboratory individuals reared in the field (Lab/Field) showed a borderline but not significant reduction in survival and mating rate. Finally, combining all data available on survival and mating success across both years showed that Field/Field and Lab/Lab individuals did not differ in survival and on average 76.3% of females and 60.5% of males survived daily. However the Field/Lab and Lab/Field survived significantly less well with 62% of females and 47.7% of males surviving per day. In terms of mating performance, no differences were found between groups in 2011 nor when combining all replicates conducted in 2010 and 2011.

Assortative mating is key to malaria control targeting local vector populations via the release of mass-produced mosquitoes [44], yet this aspect of mosquito rearing programmes has never been formally investigated. The results of this study show that laboratory-reared males and females from a laboratory strain of the Mopti form were unable to recognize their own kind and mated equally with individuals of the Savanna form in large outdoor mating cages. Under the same conditions, Field/Field males and females mated mostly assortatively and overall the Field/Lab and Lab/Field mated assortatively, although in the latter two treatment groups, sample sizes were too limited to test each sex separately.

That the overall survival and insemination rates of Lab/Lab individuals did not differ from that of Field/Field individuals is promising regarding prospective mosquito release projects and shows that colonized strains from a potential target population can perform as well as the progeny of wild-caught females under controlled environmental conditions. These results suggest that there was no loss in genetic and phenotypic quality associated with the 2–3 years of colonization and rearing process of our Mopti strain of *An. gambiae*. From a practical point-of-view, the transportation of adults to the field site inherent to the design of this study and to mosquito release programs had not apparent effect on survival and mating rates which bodes well for the future. The mean daily survival of Field/Field and Lab/Lab females was 76.3%, which lies within the 66.5–82.4 range estimated in mark-release-recapture and sporozoite rate studies of *An gambiae* sensu lato in similar, West African Sudan savanna habitats of Mali and Burkina Faso [45], [46]. These studies did not focus on male survival rate, which was equivalent here to 60.5%. In addition, they did not distinguish *An. arabiensis* from *An. gambiae s.s*. and, within this species, between the Mopti and Savanna forms. Recent hydric stress studies have shown that the two forms differ in their resistance to desiccation [47], [48] and the lower survival of Savanna females and males compared to Mopti individuals from matching cohorts observed here lends further support to those observations. It is noteworthy that shelter and access to water were intentionally limited in the semi-field cages in an attempt not to obscure potential survival differences between experimental groups. This, combined with particularly dry and hot September and October months in 2011, may have exaggerated intrinsic survival differences between Mopti and Savanna individuals under cage conditions in the assortative mating experiment.

Our experimental design further enabled us to test whether laboratory rearing at the larval stage negatively impacted the survival of field progeny and, reciprocally, if larval rearing in the field could potentially improve lab-produced individuals. Neither of these groups survived very well, suggesting that the discrepancy between the parental environmental conditions and that experienced by their progeny may have disrupted developmental processes affecting adult survival. As an example, the contrasted patterns of daily temperatures experienced by the parental generation may have resulted in maladapted patterns of metabolites storage in their transplanted larval progeny. Variation in adult body lipid, glycogen and water content are known to be crucial for resistance to desiccation hence an important determinant of survival [49]. Thus transgenerational epigenetic inheritance mechanisms could be responsible for decreased adult survival of the Field/lab and Lab/Field transplanted experimental groups, as has been shown in a number of other cross-generational transplantation studies [50]. Interestingly, these differences did not significantly negatively impact the mating success of transplanted groups that was comparable to Field/Field and Lab/Lab individuals. Generally, across all replicates and treatment groups, we found no relationship between mean female survival and their mean mating rate but mean male survival strongly correlated with female insemination rates. This broad correlation tends to support Bateman's principle - i.e. the prediction posited by sexual selection theory that variation in phenotypic quality affects the reproductive success of males more strongly than that of females [51], [52]. Males of higher phenotypic quality would enjoy higher survival and mating rate than males of poorer phenotypic quality because of female choice. Variation in phenotypic quality of females, on the other hand, would not prevent them from being mated with.

The most critical finding in this study is the lack of assortative mating observed in Lab/Lab females and males compared to the other three groups. This pattern can only be explained through complex genetic*environment interactions affecting the females' and males' adult behavioural repertoires. That Field/Lab individuals retained assortative mating choosiness is surprising and could potentially be explained through complex epigenetic effects carrying over from their previous generation as wild adults. Additionally, the assortative mating behaviour of Lab/Field individuals suggests that their larval development or pupation and emergence in the field deeply affected their mating behaviour as adults. Future studies are required to confirm and clarify these findings. Understanding their underlying mechanisms is particularly important given that they could potentially offer a solution to the deficient mating phenotypes of mass-produced laboratory-reared mosquitoes.

Average mating frequency across all replicates and experiments was 11.3% in treatment females exposed to male progeny from the field reared in the field and 16.8% in males exposed to such female progeny. This is much lower than the average 54.9% observed for wild-caught and colonized (∼3 years) *An. arabiensis* individuals in 6×8 m semi-field cages in Northern Sudan [30]. In another study conducted in 2.84×3.63 m enclosures within a greenhouse in Ohio, USA, insemination rate by 3-day old male *An. gambiae* from a 20+ year-old colony was 26.5% [53]. The same study showed that males younger than 3 days inseminated fewer females and older males ∼33% of females per night. Importantly, both studies used a 2∶1 male to female ratio whilst a 1∶1 ratio was used here in order to make use of the majority of females and males produced. In nature the sex-ratio in swarms is heavily biased towards males and females may copulate only with the best available males [54]. Thus a 1∶1 ratio might have led to a shortage of good males and thus constrained mating rates. At present, we do not know if the size of the enclosures used at N'Gabacoro Droit significantly interfered with swarming or not but the observation that laboratory-reared Mopti individuals commonly swarm in their standard 5 L cages, would tend to suggest that swarming should not pose a problem in the comparatively larger semi-field enclosures. No particular efforts were made to attempt visualizing swarms once it was established from preliminary studies that mating occurred successfully. Nevertheless, that cross-mating between the Field/Field Mopti and Savanna individuals was much more frequent than the ∼1.4% observed in wild M and S form populations from the same locale [39] suggests that our experimental conditions did not fully and perfectly reproduce the natural conditions required for complete assortative mating.

Another limitation of this study is that it focuses on the Mopti form, one of the easiest populations to colonize and maintain in the laboratory. In populations that are difficult to colonize the resulting mating phenotype of laboratory-reared individuals is more likely to have shifted away from that of wild individuals as a result of adaptation to the laboratory. As a result it is likely that insectary-produced individuals would not perform as well when compared with wild-type individuals [8]. Finally, and despite providing the first baseline data on survival and mating success of females and males from different genetic and environmental backgrounds, this experiments did not consider the mating competitiveness of Lab/Lab and Field/Field males versus wild-type males reared directly from wild-caught larvae and pupae. Thus the effect of growing in a completely natural environment on mating performance remains to be evaluated using field releases. Interestingly, that the Field/Lab individuals survived significantly less well than non-transplanted Lab/Lab and Field/Field individuals in our experiment highlights a potential flaw of past experiments that used males reared from field collected larvae reared in the laboratory against laboratory-reared ones [30], [53] suggesting that such approach might unintentionally and artificially boost the mating performance of all-laboratory reared males.

Here the good survival and mating performance of colonized Mopti females and males reared in the laboratory were mitigated by their incapacity to mate assortatively. This characteristic, albeit underlying behavioural deficiencies at the adult stage, could in some cases be used to the advantage of release strategies. In areas where complex *An. gambiae* populations occur in sympatry, the relative loss in mating efficiency inherent to releasing males that mate indiscriminately could be compensated by the advantage of targeting females of multiple cryptic taxa simultaneously. If, however, assortative mating is required as should be the case for most release projects, then a rearing facility that includes outdoor larval rearing and adult emergence processes may prove critical. Future studies will need to establish whether those requirements are specific to the Mopti form or may apply to all cryptic taxa of the *An. gambiae* complex.

## Materials and Methods

### Mosquito Colonisation

All experiments were conducted at the Malaria Research and Training Centre (MRTC), University of Bamako, Bamako, Mali during the rainy seasons (July–October) of 2010 and 2011. An initial colony of Mopti form of *An. gambiae* s.s. was established in 2009 from the progeny of gravid females collected in the village of N'Gabakoro Droit (12°39′46″N, 7°50′34″W) and maintained in an insectary at the MRTC. Mosquito captures were discussed and authorized by the local authorities (village chief Zoumana Doumbia). At the start of the 2010 experiment this lab colony had reached generation F42, and was well adapted to laboratory maintenance including feeding on membrane feeders.

### Insectary environment

Laboratory-based mosquito rearing and maintenance took place in the insectary of the recently-build biosafety-level 3 Transgenic Mosquito Laboratory designed to house the project sponsored by Wellcome Trust programme grant. The insectary features a glass-brick wall thus providing a natural day-dusk-night-dawn light cycle. Air temperature was maintained at a constant 27±2°C and relative humidity was kept at 70±5% at all times and the water temperature in larval growth trays was 22.5±0.5°C - although this was not actively regulated. Adult mosquitoes were maintained in 5 L cylindrical polypropylene bucket (∼20.5 cm height×20 cm diameter) with a sleeved side opening and netting top and provided with 10% sucrose solution and H_2_O *ad libitum*.

### Field cages environment

Field-based mosquito rearing and mating experiments were conducted in four 4×4×2 m and one 2×2×2 m custom-made nylon netting (1 mm weave mesh) enclosures (Howitec, Bolsward, The Netherlands) supported by a timber frame and covered with a tarpaulin roof (GalaTent Rotherham, UK). These were built along a North-South axis in a grassy clearing in the outskirts of the village of Banankoro in the outskirts of the locality of N'Gabacoro Droit (12°39′46″N, 7°50′34″W). The location of the outdoor cages for mosquito studies was discussed and authorized by the local authorities (village chief Hady Diarra). The site is bordered on the North by a breezeblock wall and the enclosures were positioned so as to receive comparable levels of sunshine and shade. Each enclosure possessed an antechamber to prevent mosquitoes from entering or escaping the cages. Three of the 4×4×2 m enclosures were used exclusively for mating and survival studies, with the fourth acting as an insectary in 2010 and as additional experimental enclosure in 2011. A smaller 2×2×2 m enclosure was used as insectary and field lab in 2011.

Mating enclosures were provided with a 3 cm deep floor-covering of coarse fluvial gravel kept moist to enhance humidity, 3 large (30×80 cm) cylindrical clay pots with a 10 cm deep layer of wet gravel providing shaded and humid resting sites, and two large leafy plants to provide shade and additional humidity through transpiration in the cage. During each experimental period each plant had several cotton wool pads soaked with 10% sucrose solution attached to it to mimic flowers and provide an energy source. Air temperature at the field site reached daytime highs of 34–42°C and night lows of 24–28°C. Water temperature in the larval growth trays ranged from 24–32°C over the course of a typical 24 h period, with the highest temperature recorded at 36°C. Relative humidity within the field enclosures was between 40 and 80%. Daytime temperatures within the clay pot refuges in each mating enclosure were consistently 4–5°C below air temperature and RH between 60 and 80%.

### Production of progeny from colony and field genetic background

‘Lab’ progeny were obtained by blood feeding the colonized strain and, after allowing 48 hrs for egg development, providing it with an oviposition pot (50 ml polystyrene cup) containing moistened filter paper. Forty-eight hours later the oviposition cup was removed and the eggs placed in a rearing tray filled with 1 l H_2_O in order to hatch.

In order to provide ‘field’ progeny throughout the experiments, field captures of gravid females were carried out using mouth aspirators in huts from the village of Banankoro. Mosquito captures were discussed and authorized by the local authorities (village chief Hady Diarra). Captured mosquitoes were then transferred to a 5 l cage and provided with a 10% sucrose solution and H_2_O *ad libitum* and transported by car to the insectary at the MRTC. Forty-eight hours after capture; individual females were transferred to individual oviposition tubes. After egglaying, individual egg batches were transferred to 15 ml H_2_O in a 25 ml weigh boat for hatching and provided with a suspension of yeast cells (Liquifry, Interpet, Dorkin, United Kingdom) until females were genotyped. DNA extractions from females were carried out using DNAzol (Invitrogen, Carlsbad, CA, USA). The PCR/RFLP diagnostic developed by Fanello *et al.* [55] was used to differentiate *An. gambiae s.s.* females from those belonging the sister species *An. arabiensis*. The same diagnostic also indicated which individuals belonged to the M and S molecular forms amongst *An. gambiae* s.s., In Mali M-form individuals belong to the Mopti chromosomal form whilst S-form ones can be belong to the Savanna or Bamako chromosomal forms [35], [36], [37]. Once successfully genotyped, Mopti M-form *An. gambiae* s.s. broods (1st instar larvae) were pooled and prepared for rearing in the indoors insectary or to be transported to the field site. S-form broods were further characterised to determine if they belonged to the Savanna or Bamako chromosomal form using the PCR diagnostic based on the *J*-inversion polymorphism developed by Coulibaly *et al.* [56]. Once identified, Savanna broods were pooled and prepared for transport to the field site. *An. arabiensis* and Bamako broods were discarded.

### Larval rearing under ‘lab’ and ‘field’ conditions

First instar larval progeny from the laboratory background females or from field-caught females were either reared in the indoors insectary at a density of 200 larvae in 1 L H_2_O in standard rearing trays. These constituted our Lab/Lab and Field/Lab experimental groups. Alternatively, first instar larvae were transported by car to the field site in a 1 L of H_2_O in a glass Duran bottle. Following transport, these larvae were similarly distributed in rearing trays set on the floor of the outdoor cage used as field insectary. These constituted our Lab/Field and Field/Field experimental groups. Thus the combination of two genetic backgrounds and two rearing environments resulted in 4 Gen/Env experimental groups.

The number of trays set up for each group varied depending on the number of adults required for each experiment. In the outdoor insectary, larval trays were stored at or near ground level in an effort to provide a natural horizon for developing larvae. During development through the L1–L4 larval instars, all larvae were initially supplied with a yeast cell suspension (Liquifry, Interpet, Dorkin, United Kingdom) followed by a standardized regimen of ground fish food (Tetramin, Tetra, Melle, Germany). Upon pupation, pupae were sexed using a binocular dissecting microscope (Leica, Wetzlar, Germany) and transferred by aspiration to small polystyrene cups. In the lab, pupae were set to emerge and kept in standard 5 L rearing cages until they were transported to the field insectary for mating experiments (30′ drive). In the field insectary, sexed pupae were placed into larger holding enclosures for emergence. Holding cages were covered with a thick layer of wet humid paper towels in order to maintain 60–80% RH at all times for newly emerged imagines. Imagines at the lab or field site were supplied with a 10% sucrose solution and H_2_O *ad libitum* at all times.

### Experimental procedure 1st experiment - Genetic/Environmental effects on survival and mating success within form

A first experiment was conducted over 2010–2011 in order to assess the effects of colonisation (genetic background) and the larval rearing conditions (environmental background) on survival and mating success within the Mopti form population. As described above, mosquitoes from the laboratory or field background were reared in the lab or field insectary resulting in four experimental groups (Field/Field, Field/Lab, Lab/Field and Lab/Lab). Due to equipment constrains (3 mating enclosures available in 2010) this experiment was split into two and carried out over both the 2010 and 2011 rainy seasons. In 2010 we compared the Field/Field, Field/Lab and Lab/Lab groups. Three replicates of this comparison focused on females from the 3 treatment groups that were all given field/field males to mate with, and 3 other replicates focused on the reciprocal experiments, this time focusing on treatment males. In August–September 2011, we investigated the Field/Field, Lab/Field and Lab/Lab groups using identical procedures. This split over two years was accounted for statistically by using a nested design (see statistical procedures).

Experimental procedures were identical for both field seasons. For the Lab/Lab and Field/Lab groups, samples of 50 virgin adults 3–5day-old were collected at random by aspiration from the main cages in the indoor insectary and placed in standard 5l polypropylene cages. The cages were brought to the field by car (30′ drive) and left to acclimatise in the outdoor insectary before the start of the mating experiment. In the field, imagines for the Field/Field group were collected at random from the holding enclosures kept in the field insectary. Three additional samples of 50 Field/Field individuals of the opposite sex were similarly prepared. All 5 L cages were provided with water and sugar solution and left to rest for a minimum of 2 h before being released. At ∼1700 h local time each treatment cage was paired up with a Field/Field cage of the opposite sex and the imagines released into the large experimental mating enclosures. After ∼40 h (2 nights) surviving individuals were recaptured from within their enclosures using a large backpack aspirator (JW Hock & Co, Gainsville, FL, USA). Three sweeps over ∼2 h were carried out in order to recapture the majority of surviving individuals from the 3 enclosures. The experimental enclosures used for each cross were rotated between 3 replicates of the experiment in order to account for the effect of environmental variation between enclosures. In the few days that separated each replicate, neither sugar water nor water were provided in the enclosures to ensure that any uncaptured mosquito died before the next replicate. For each field season, these experimental procedures resulted in 18 experimental crosses being conducted - i.e. 3 experimental groups, 3 replicates, and reciprocal crosses focusing either on treatment females or males - for a total of 3600 mosquitoes.

Recaptured individuals were transported back to the lab at the MRTC. The body size of both male and female individuals was measured as length of the wing from the posterior anal cell margin to the tip of radial vein 3 at 20× magnification. Females were stored at −20°C in 70% ethanol for at least 24 h before being dissected to ascertain their mating status based on the presence of absence of a sperm bundle within their spermatheca.

### Experimental procedure 2nd experiment - Genetic/Environmental effects on survival and assortative mating

A second experiment was conducted in 2011 in order to study the effects of colonisation (genetic background) and larval rearing conditions (environmental background) on assortative mating between forms [39]. The experiment was carried out entirely in 2011 and used the same experimental procedures. A fourth large outdoor cage was set-up, thus enabling to compare all 4 experimental groups (Field/Lab, Lab/Field, Lab/Lab and Field/Field). In this experiment, virgin adults of the four M-form treatments were mated with an equal number of a 1∶1 mix of Field/Field Mopti form and Savanna form individuals of the opposite sex. A total of 24 crosses - i.e. 4 experimental groups, 3 replicates, and reciprocal crosses focusing on females and males - were conducted involving 2400 mosquitoes.

Following the two nights of mating, the body size of recaptured individuals (wing length) was measured. The genotype of all survivors was determined by PCR/RFLP (see above). In addition, the mating status of recaptured females was determined as described previously. Further, the genotype of the male that inseminated a given female was determined by PCR analysis of transferred sperm as done in previous studies [39], [57]. Briefly, once isolated by dissection, the sperm bundle was rinsed in a clean drop of water and DNA was isolated from it using the ChargeSwitch DNA extraction kit protocol (Invitrogen, Carlsbad, CA, USA). The molecular form of the transferred sperm was then determined by PCR/RFLP [55].

### Statistical procedures

All statistical analyses were performed using the software JMP9.0 (SAS Institute, Inc). All continuous data were checked for normality and heteroscedasticity.

In the first experiment conducted over the 2010–2011 field seasons, general linear models were used to test the effect of the independent variable ‘Gen/Env’ (i.e. combination of genetic and environmental backgrounds) on male and female body size and included the covariates ‘year’ and ‘replicate’ whenever relevant to the biological question under investigation. Interactions and the direct effect of replicate were tested but reported only when significant. Non-significant interactions were removed from models using a step-wise procedure followed by non-significant direct effects. Model effects were nested by year to account for the fact that the experiment was carried out over two seasons.

Nominal logistic regressions were used to test the effect of the independent variable ‘Gen/Env’ on the dependent variables: ‘survival’ (proportion of males and females surviving), ‘mating rate’ (proportion of females inseminated), and ‘assortative mating rate’. Logistic regression models included the covariates: ‘year’, ‘replicate’, and ‘enclosure’, whenever relevant to the biological question under investigation. Interactions and the direct effect of replicate were tested but reported only when significant. Non-significant interactions were removed from models using a step-wise procedure followed by non-significant direct effects. Model effects were nested by year to account for the fact that the experiment was carried out over two field seasons.

In the second experiment, conducted entirely in 2011 using 4 enclosures, non-nested general linear models and nominal regressions were used using a similar step-wise procedure in order to test the effects of the ‘Gen/Env’ treatment, ‘replicate’ and ‘enclosure’ on body size, and survival, mating, and assortative mating rates respectively.

Post-hoc pairwise comparisons between treatment groups were conducted using the Marascuilo procedure for frequencies and Tukey-tests for continuous data.
